# Defective Ventilation of Hospitals
*On the Defects with reference to the Plan of Construction and Ventilation of most of our Hospitals for the reception of the Sick and Wounded. By John Roberton, Surgeon. Manchester: 1856.


**Published:** 1856-07

**Authors:** 


### THE EPITOME OF SANITARY LITERATURE.

### DEFECTIVE VENTILATION OF HOSPITALS.*

NOT long ago, says Mr. Roberton, I had occasion to inspect the dormitories in a school, in each of which fourteen boys had for some time slept, with a cubic air-space for each boy of barely 140 feet. Ere long the ill effects appeared; seven of the boys and two of the masters sickened of typhus, and the school had for a time to be broken up.

Dr. Mackinnon, in his work on the *Public Health of Bengal*, published in 1848, states that the number of prisoners in the gaols is usually about 40,000, chiefly natives; the average mortality annually of these had recently been one in ten, rising in some instances to more than one in four, viz., to 26 per cent. This rate of mortality is accounted for, on the fact that in no instance is there an allowance of more than 300 cubic feet of space for each individual, whilst in some instances 70 cubic feet is the "murderous" average. From 800 to 1,000 cubic feet is the smallest possible amount of air for one individual; with this calculation we entirely concur.

As regards the construction of hospitals for the sick and wounded, Mr. Roberton makes a remark—a most common-sense and original remark. "We have been," he says, "in the habit of confounding two things widely different, viz., sick wards and dormitories. It is owing to ignorance or inattention to this essential difference that we have few hospitals in England that are not insalubrious whenever they are crowded; and which, when crowded with such cases as burns, compound fractures, and extensive ulcers, are often the abodes of deaths occurring in forms most humbling and mortifying to the pride of surgical science. The air-space for each bed in a number of our provincial hospitals is not half what it should be."

Another defect to which Mr. Roberton refers with great force is, that in English hospitals there are no arrangements to prevent the foul air of one ward from becoming diffused over the whole of the building—thus polluting the whole. On the continent, however, this all important preventive process is attained in many instances, but in none so completely as in the *Hospital of Bordeaux*.

The construction of the Bordeaux hospital is simple, effec-

* On the Defects with reference to the Plan of Construction and Ventilation of most of our Hospitals for the reception of the Sick and Wounded. By JOHN ROBERTON, Surgeon. Manchester: 1856.

tive, elegant. Its main advantage is, that no single ward communicates with another one. Passing into the hospital by the centre gateway, the visitor beholds a beautiful court, and on the two sides facing each other a succession of pavilions or tiers of building, standing parallel and having the ends towards the square. The tiers of building are separated from each other by a neat and elegant garden, and in these tiers are the sick wards of the place, each isolated and independent.

Each ward is about 140 feet long, 30 feet wide, and 20 feet high. It has tall narrow windows on each side, facing the opposite ward, and contains thirty-eight beds, nineteen in a row. The bedsteads, small and of iron, are hung round with dimity curtains, but are without the tester. Free ventilation is secured through the windows, and the look out on either side is into a beautiful garden, fifty feet broad. The ward has only one door, which serves both for entrance and for exit, and this leads into the open air. The wards in the upper stories are reached by a staircase without. Thus every ward is in fact a separate hospital, and the foul air from the one cannot enter into the other, because it passes direct into the *external* atmosphere and is there diffused.

A peculiar advantage connected with this arrangement is, that there is no limit to the size of the hospital or the number of beds. In England, the larger the hospital is, the higher is the rate of mortality, because the construction is after the manner of an hotel, with each room subjected to the influence of the air of the other rooms. In the Bordeaux hospital there are 728 beds.

On the subject of ventilation, Mr. Roberton remarks that he has little faith in so-called "scientific ventilation." "I should almost as soon think," he tells us, "of looking for my daily supply of water for ablution from the scientific formation of it out of its aeriform elements by galvanic agency, as upon depending upon such refined expedients for the purity of the sick ward. If a ward is to be kept perfectly sweet, the air must flow through it in correspondence with the natural movements of the atmosphere without. Let the windows of the opposite side walls (they ought to reach near to the ceiling) be thrown open, and instantly the air enters at one side and escapes at the other, the side of admission being determined by the direction of the wind at the time. There is an unceasing flow of the atmosphere, mostly parallel to the surface of the earth, which fans and purifies everything. A perfectly stagnant condition of the atmosphere—if it ever exist—is extremely rare." Curious and refined modes of ventilation therefore, says this writer, fail when applied to a hospital, the effluvia of which can be dissipated by no ventilation other than nature's—the ceaseless, it may be imperceptible, flow of the external air through the wards.

We gather from the rest of Mr. Roberton's Treatise, which, like all his writings, is remarkable for clear common sense and fearless expression of the truth, the following rules.

The windows of hospital walls should be "tall;" they should approach the ceiling.

The annoyance of "draughts" may be prevented by admitting the air current through perforated zinc.

Beyond fireplaces and open stoves, artificial modes of heating are injurious. Heat promotes the decomposition of the excretions; and we cannot increase the discomfort of patients more than by surrounding them with an unnaturally dry, hot atmosphere.

In every ward there should be a discharging shaft in the wall, shut by a sliding cover, by which soiled clothes, fouled dressings, and the like, may be removed at once to the wash cellar.

The ceiling and walls should be painted and highly varnished, so that they do not imbibe effluvia.

There should be no unnecessary furniture in the ward.

There should be a separate eating room for convalescents.

There should be every attention paid to cleanliness, not only of the ward itself, but of the persons of the patients and their attendants.

The site of a hospital ought to be an elevation to the windward of a city; on a soil naturally dry, or admitting of easy drainage; while the plot of ground ought to be large enough to include gardens, not merely to please the eye of the sick, but to provide as well the means of recreation and exercise for convalescents.

There should be accident rooms in the streets of all our larger towns as there are in Paris, where sufferers may be conveyed and promptly attended to, instead of their being removed, it may be, to a remote hospital before the wound is dressed, the dislocation reduced, or the broken bone adjusted.

